# *N*-(3,4-Dimethyl­phen­yl)benzene­sulfonamide

**DOI:** 10.1107/S1600536810026656

**Published:** 2010-07-10

**Authors:** Peter John, Shahzad Sharif, Islam Ullah Khan, Saima Khizar, Edward R. T. Tiekink

**Affiliations:** a Materials Chemistry Laboratory, Department of Chemistry, Government College, University, Lahore 54000, Pakistan; b Department of Chemistry, University of Malaya, 50603 Kuala Lumpur, Malaysia

## Abstract

The structure of the title compound, C_14_H_15_NO_2_S, shows the sulfonamide N atom to be approximately perpendicular to the plane through the S-bound benzene ring [the N—S—C—C torsion angle is −87.4 (3)°] and to lie to the opposide side of this ring to the two sulfonamide O atoms. The N-bound benzene ring is splayed out with respect to the rest of the mol­ecule so that overall, the mol­ecule adopts a twisted conformation. The dihedral angle between the two benzene rings is 64.5 (3)°. In the crystal, supra­molecular chains aligned along the *b* axis are formed *via* N—H⋯O hydrogen bonds.

## Related literature

For background to the pharmacological uses of sulfonamides, see: Korolkovas (1988 ▶); Mandell & Sande (1992 ▶). For the structure of the *S*-tosyl derivative, see: Gowda *et al.* (2009 ▶). For related structures, see: Khan *et al.* (2010 ▶); Sharif *et al.* (2010 ▶).
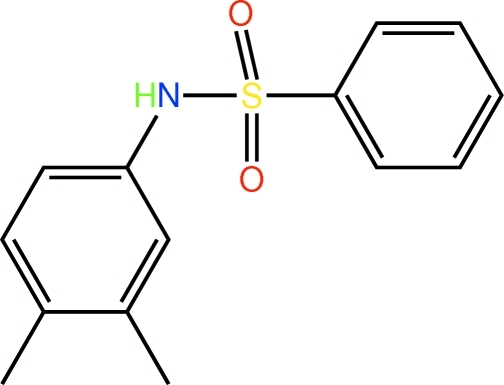


## Experimental

### 

#### Crystal data

C_14_H_15_NO_2_S
*M* *_r_* = 261.33
Monoclinic, 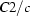

*a* = 24.2129 (12) Å
*b* = 9.2616 (5) Å
*c* = 16.5584 (16) Å
β = 132.014 (2)°
*V* = 2758.9 (3) Å^3^
*Z* = 8
Mo *K*α radiation
μ = 0.23 mm^−1^
*T* = 293 K
0.31 × 0.08 × 0.07 mm

#### Data collection

Bruker APEXII CCD diffractometer
Absorption correction: multi-scan (*SADABS*; Sheldrick, 1996 ▶) *T*_min_ = 0.795, *T*_max_ = 0.947
11486 measured reflections
2864 independent reflections
2112 reflections with *I* > 2σ(*I*)
*R*_int_ = 0.036

#### Refinement

*R*[*F* ^2^ > 2σ(*F* ^2^)] = 0.055
*wR*(*F* ^2^) = 0.196
*S* = 1.09
2864 reflections
168 parameters
37 restraints
H atoms treated by a mixture of independent and constrained refinement
Δρ_max_ = 0.56 e Å^−3^
Δρ_min_ = −0.65 e Å^−3^

### 

Data collection: *APEX2* (Bruker, 2007 ▶); cell refinement: *SAINT* (Bruker, 2007 ▶); data reduction: *SAINT*; program(s) used to solve structure: *SHELXS97* (Sheldrick, 2008 ▶); program(s) used to refine structure: *SHELXL97* (Sheldrick, 2008 ▶); molecular graphics: *ORTEP-3* (Farrugia, 1997 ▶) and *DIAMOND* (Brandenburg, 2006 ▶); software used to prepare material for publication: *publCIF* (Westrip, 2010 ▶).

## Supplementary Material

Crystal structure: contains datablocks global, I. DOI: 10.1107/S1600536810026656/hb5533sup1.cif

Structure factors: contains datablocks I. DOI: 10.1107/S1600536810026656/hb5533Isup2.hkl

Additional supplementary materials:  crystallographic information; 3D view; checkCIF report

## Figures and Tables

**Table 1:** Hydrogen-bond geometry (Å, °)

*D*—H⋯*A*	*D*—H	H⋯*A*	*D*⋯*A*	*D*—H⋯*A*
N1—H1*n*⋯O2^i^	0.85 (3)	2.07 (2)	2.921 (3)	177 (5)

Symmetry code: (i) 
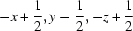
.

**Fig. 1.**
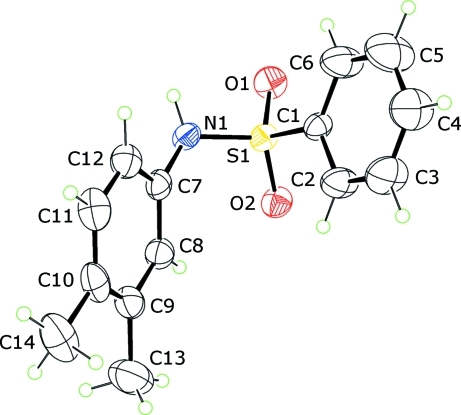
The molecular structure of (I) showing displacement ellipsoids at the 35% probability level.

**Fig. 2.**
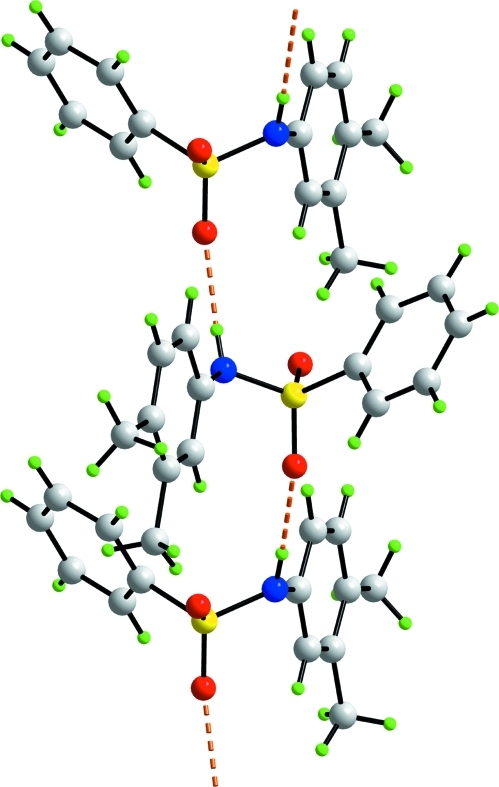
A view of the supramolecular chain mediated by N–H···O hydrogen bonding (orange dashed lines) in (I). Colour code: S, yellow; O, red; N, blue; C, grey; and H, green.

## supplementary crystallographic information

### Comment

In continuation of on-going structural studies of sulfonamides (Khan *et al.*, 2010; Sharif *et al.*, 2010), of interest owing to their putative anti-microbial activity (Korolkovas, 1988; Mandell & Sande, 1992), the crystal and molecular structure of the title compound, (I), was investigated.

In (I), both sulfonamido-O atoms lie to one side of the S-bound benzene ring [the O1–S1–C1–C6 and O2–S1–C1–C2 torsion angles are 19.3 (4) and -28.0 (3) °, respectively] and are on the opposite side of this ring to the sulfonamido-N1 atom, Fig. 1. The latter is approximately perpendicular to the benzene ring [N1–S1–C1–C2 is -87.4 (3) °] with the N-bound benzene ring clearly displaced to one side of the molecule; the dihedral angle formed between the two benzene rings is 64.5 (3) °. Although not isomorphous, the overall molecular conformation found in the closely related derivative, with an S-bound tolsyl group, is almost identical (Gowda *et al.* 2009).

The presence of N1–H···O2 hydrogen bonding, Table 1, leads to the formation of supramolecular chains along the *b* axis, Fig. 2.

### Experimental

To 3,4-dimethyl aniline (484 mg, 4 mmol) in distilled water (10 ml) was added benzene sulfonyl chloride (510 ml, 4 mmol) with stirring at room temperature while maintaining the pH of the reaction mixture at 8 using 3% sodium carbonate. The progress of the reaction was monitored by TLC. The precipitate formed was washed with water, dried and crystallized from a methanol/ethyl acetate mixture (50:50 V/V) to yield colourless prisms of (I); m. pt 414 K.

### Refinement

The C-bound H atoms were geometrically placed (C–H = 0.93–0.96 Å) and refined as riding with *U_iso_*(H) = 1.2*U_eq_*(C). The N-bound H atom was refined with the distance restraint N–H = 0.86±0.01 Å, and with *U_iso_*(H) = 1.2*U_eq_*(N). High thermal motion was noted for several atoms in the S-bound benzene ring but multiple positions were not resolved. The anisotropic displacement parameters for this ring were refined with the ISOR command to constrain these to be approximately isotropic.

### Crystal data

**Table:**

C_14_H_15_NO_2_S	*F*(000) = 1104
*M**_r_* = 261.33	*D*_x_ = 1.258 Mg m^−^^3^
Monoclinic, *C*2/*c*	Mo *K*α radiation, λ = 0.71073 Å
Hall symbol: -C 2yc	Cell parameters from 2975 reflections
*a* = 24.2129 (12) Å	θ = 2.5–24.2°
*b* = 9.2616 (5) Å	µ = 0.23 mm^−^^1^
*c* = 16.5584 (16) Å	*T* = 293 K
β = 132.014 (2)°	Prism, colourless
*V* = 2758.9 (3) Å^3^	0.31 × 0.08 × 0.07 mm
*Z* = 8	

### Data collection

**Table:**

Bruker APEXII CCD diffractometer	2864 independent reflections
Radiation source: fine-focus sealed tube	2112 reflections with *I* > 2σ(*I*)
graphite	*R*_int_ = 0.036
φ and ω scans	θ_max_ = 26.5°, θ_min_ = 2.3°
Absorption correction: multi-scan (*SADABS*; Sheldrick, 1996)	*h* = −30→30
*T*_min_ = 0.795, *T*_max_ = 0.947	*k* = −11→11
11486 measured reflections	*l* = −20→20

### Refinement

**Table:**

Refinement on *F*^2^	Primary atom site location: structure-invariant direct methods
Least-squares matrix: full	Secondary atom site location: difference Fourier map
*R*[*F*^2^ > 2σ(*F*^2^)] = 0.055	Hydrogen site location: inferred from neighbouring sites
*wR*(*F*^2^) = 0.196	H atoms treated by a mixture of independent and constrained refinement
*S* = 1.09	*w* = 1/[σ^2^(*F*_o_^2^) + (0.1221*P*)^2^ + 0.6451*P*] where *P* = (*F*_o_^2^ + 2*F*_c_^2^)/3
2864 reflections	(Δ/σ)_max_ < 0.001
168 parameters	Δρ_max_ = 0.56 e Å^−^^3^
37 restraints	Δρ_min_ = −0.65 e Å^−^^3^

### Special details

**Table:**

Geometry. All s.u.'s (except the s.u. in the dihedral angle between two l.s. planes) are estimated using the full covariance matrix. The cell s.u.'s are taken into account individually in the estimation of s.u.'s in distances, angles and torsion angles; correlations between s.u.'s in cell parameters are only used when they are defined by crystal symmetry. An approximate (isotropic) treatment of cell s.u.'s is used for estimating s.u.'s involving l.s. planes.
Refinement. Refinement of *F*^2^ against ALL reflections. The weighted *R*-factor *wR* and goodness of fit *S* are based on *F*^2^, conventional *R*-factors *R* are based on *F*, with *F* set to zero for negative *F*^2^. The threshold expression of *F*^2^ > 2σ(*F*^2^) is used only for calculating *R*-factors(gt) *etc*. and is not relevant to the choice of reflections for refinement. *R*-factors based on *F*^2^ are statistically about twice as large as those based on *F*, and *R*- factors based on ALL data will be even larger.

### Fractional atomic coordinates and isotropic or equivalent isotropic displacement parameters (Å^2^)

**Table:**

	*x*	*y*	*z*	*U*_iso_*/*U*_eq_	
S1	0.21969 (4)	0.08206 (8)	0.26474 (6)	0.0437 (3)	
O1	0.16507 (13)	0.0323 (3)	0.15598 (16)	0.0610 (6)	
O2	0.22892 (12)	0.2337 (2)	0.28664 (18)	0.0567 (6)	
N1	0.29812 (14)	0.0196 (3)	0.3083 (2)	0.0492 (6)	
H1n	0.291 (2)	−0.063 (2)	0.280 (3)	0.059*	
C1	0.20212 (16)	0.0052 (3)	0.3427 (2)	0.0448 (7)	
C2	0.2297 (2)	0.0696 (4)	0.4375 (3)	0.0690 (10)	
H2	0.2586	0.1526	0.4613	0.083*	
C3	0.2149 (3)	0.0117 (5)	0.4979 (3)	0.0875 (13)	
H3	0.2337	0.0557	0.5625	0.105*	
C4	0.1730 (3)	−0.1089 (5)	0.4635 (4)	0.0935 (14)	
H4	0.1643	−0.1497	0.5054	0.112*	
C5	0.1437 (4)	−0.1704 (6)	0.3671 (5)	0.1114 (18)	
H5	0.1129	−0.2507	0.3418	0.134*	
C6	0.1591 (3)	−0.1147 (5)	0.3065 (4)	0.0851 (13)	
H6	0.1403	−0.1588	0.2420	0.102*	
C7	0.36691 (17)	0.0393 (3)	0.4183 (2)	0.0470 (7)	
C8	0.39442 (19)	0.1756 (3)	0.4603 (3)	0.0537 (8)	
H8	0.3673	0.2562	0.4178	0.064*	
C9	0.46209 (19)	0.1938 (4)	0.5652 (3)	0.0607 (9)	
C10	0.50388 (19)	0.0737 (4)	0.6287 (3)	0.0638 (10)	
C11	0.4750 (2)	−0.0623 (4)	0.5834 (3)	0.0683 (10)	
H11	0.5022	−0.1436	0.6246	0.082*	
C12	0.4080 (2)	−0.0805 (4)	0.4804 (3)	0.0581 (8)	
H12	0.3902	−0.1728	0.4523	0.070*	
C13	0.4905 (3)	0.3462 (5)	0.6065 (4)	0.1030 (16)	
H13A	0.5075	0.3559	0.6778	0.154*	
H13B	0.4511	0.4139	0.5581	0.154*	
H13C	0.5309	0.3653	0.6096	0.154*	
C14	0.5778 (2)	0.0900 (6)	0.7430 (3)	0.0921 (15)	
H14A	0.6053	0.1672	0.7454	0.138*	
H14B	0.6054	0.0017	0.7654	0.138*	
H14C	0.5697	0.1115	0.7912	0.138*	

### Atomic displacement parameters (Å^2^)

**Table:**

	*U*^11^	*U*^22^	*U*^33^	*U*^12^	*U*^13^	*U*^23^
S1	0.0524 (5)	0.0421 (5)	0.0424 (4)	0.0023 (3)	0.0340 (4)	0.0028 (3)
O1	0.0623 (14)	0.0769 (16)	0.0388 (12)	−0.0017 (12)	0.0317 (11)	−0.0011 (10)
O2	0.0678 (14)	0.0410 (12)	0.0680 (14)	0.0076 (10)	0.0483 (13)	0.0091 (10)
N1	0.0572 (15)	0.0462 (14)	0.0533 (15)	−0.0004 (12)	0.0407 (13)	−0.0075 (12)
C1	0.0560 (16)	0.0416 (15)	0.0466 (15)	−0.0004 (12)	0.0384 (14)	−0.0012 (12)
C2	0.089 (2)	0.072 (2)	0.0622 (19)	−0.0237 (18)	0.0574 (19)	−0.0184 (16)
C3	0.118 (3)	0.102 (3)	0.071 (2)	−0.024 (2)	0.075 (2)	−0.019 (2)
C4	0.132 (3)	0.098 (3)	0.091 (3)	−0.025 (3)	0.092 (3)	−0.005 (2)
C5	0.155 (4)	0.101 (3)	0.118 (3)	−0.054 (3)	0.108 (3)	−0.021 (2)
C6	0.121 (3)	0.079 (2)	0.084 (2)	−0.038 (2)	0.080 (2)	−0.026 (2)
C7	0.0513 (16)	0.0521 (17)	0.0538 (17)	0.0004 (13)	0.0418 (15)	0.0004 (14)
C8	0.0551 (18)	0.0536 (19)	0.0588 (19)	0.0010 (14)	0.0408 (17)	−0.0001 (14)
C9	0.0553 (19)	0.071 (2)	0.064 (2)	−0.0095 (17)	0.0436 (18)	−0.0090 (18)
C10	0.0481 (19)	0.094 (3)	0.058 (2)	−0.0018 (18)	0.0389 (17)	0.0061 (19)
C11	0.062 (2)	0.075 (3)	0.074 (2)	0.0142 (18)	0.048 (2)	0.0238 (19)
C12	0.061 (2)	0.0554 (19)	0.067 (2)	0.0035 (15)	0.0462 (18)	0.0068 (16)
C13	0.078 (3)	0.098 (4)	0.091 (3)	−0.020 (3)	0.039 (3)	−0.022 (3)
C14	0.058 (2)	0.142 (4)	0.068 (3)	−0.008 (2)	0.038 (2)	0.011 (3)

### Geometric parameters (Å, °)

**Table:**

S1—O1	1.420 (2)	C7—C8	1.379 (4)
S1—O2	1.430 (2)	C7—C12	1.381 (4)
S1—N1	1.621 (3)	C8—C9	1.386 (5)
S1—C1	1.758 (3)	C8—H8	0.9300
N1—C7	1.436 (4)	C9—C10	1.394 (5)
N1—H1n	0.85 (3)	C9—C13	1.518 (6)
C1—C6	1.356 (5)	C10—C11	1.392 (5)
C1—C2	1.364 (4)	C10—C14	1.508 (5)
C2—C3	1.379 (5)	C11—C12	1.365 (5)
C2—H2	0.9300	C11—H11	0.9300
C3—C4	1.351 (6)	C12—H12	0.9300
C3—H3	0.9300	C13—H13A	0.9600
C4—C5	1.363 (6)	C13—H13B	0.9600
C4—H4	0.9300	C13—H13C	0.9600
C5—C6	1.384 (6)	C14—H14A	0.9600
C5—H5	0.9300	C14—H14B	0.9600
C6—H6	0.9300	C14—H14C	0.9600
			
O1—S1—O2	119.67 (15)	C12—C7—N1	119.2 (3)
O1—S1—N1	105.54 (14)	C7—C8—C9	120.7 (3)
O2—S1—N1	107.46 (13)	C7—C8—H8	119.7
O1—S1—C1	108.80 (14)	C9—C8—H8	119.7
O2—S1—C1	106.57 (13)	C8—C9—C10	120.1 (3)
N1—S1—C1	108.39 (14)	C8—C9—C13	118.5 (4)
C7—N1—S1	122.54 (19)	C10—C9—C13	121.4 (4)
C7—N1—H1N	117 (2)	C11—C10—C9	117.8 (3)
S1—N1—H1N	109 (2)	C11—C10—C14	120.9 (4)
C6—C1—C2	120.5 (3)	C9—C10—C14	121.3 (4)
C6—C1—S1	119.9 (2)	C12—C11—C10	122.2 (3)
C2—C1—S1	119.6 (2)	C12—C11—H11	118.9
C1—C2—C3	120.0 (4)	C10—C11—H11	118.9
C1—C2—H2	120.0	C11—C12—C7	119.5 (3)
C3—C2—H2	120.0	C11—C12—H12	120.2
C4—C3—C2	120.1 (4)	C7—C12—H12	120.2
C4—C3—H3	120.0	C9—C13—H13A	109.5
C2—C3—H3	120.0	C9—C13—H13B	109.5
C3—C4—C5	119.7 (4)	H13A—C13—H13B	109.5
C3—C4—H4	120.1	C9—C13—H13C	109.5
C5—C4—H4	120.1	H13A—C13—H13C	109.5
C4—C5—C6	120.7 (4)	H13B—C13—H13C	109.5
C4—C5—H5	119.7	C10—C14—H14A	109.5
C6—C5—H5	119.7	C10—C14—H14B	109.5
C1—C6—C5	119.0 (4)	H14A—C14—H14B	109.5
C1—C6—H6	120.5	C10—C14—H14C	109.5
C5—C6—H6	120.5	H14A—C14—H14C	109.5
C8—C7—C12	119.7 (3)	H14B—C14—H14C	109.5
C8—C7—N1	121.0 (3)		
			
O1—S1—N1—C7	−176.1 (2)	C4—C5—C6—C1	−2.1 (9)
O2—S1—N1—C7	55.1 (3)	S1—N1—C7—C8	−60.8 (4)
C1—S1—N1—C7	−59.7 (3)	S1—N1—C7—C12	123.0 (3)
O1—S1—C1—C6	19.3 (4)	C12—C7—C8—C9	−1.6 (4)
O2—S1—C1—C6	149.6 (3)	N1—C7—C8—C9	−177.7 (3)
N1—S1—C1—C6	−95.0 (4)	C7—C8—C9—C10	1.4 (5)
O1—S1—C1—C2	−158.3 (3)	C7—C8—C9—C13	178.7 (3)
O2—S1—C1—C2	−28.0 (3)	C8—C9—C10—C11	−0.5 (5)
N1—S1—C1—C2	87.4 (3)	C13—C9—C10—C11	−177.8 (4)
C6—C1—C2—C3	0.9 (6)	C8—C9—C10—C14	179.9 (3)
S1—C1—C2—C3	178.6 (3)	C13—C9—C10—C14	2.6 (5)
C1—C2—C3—C4	0.1 (7)	C9—C10—C11—C12	−0.2 (5)
C2—C3—C4—C5	−2.1 (9)	C14—C10—C11—C12	179.4 (3)
C3—C4—C5—C6	3.1 (10)	C10—C11—C12—C7	−0.1 (5)
C2—C1—C6—C5	0.1 (7)	C8—C7—C12—C11	0.9 (5)
S1—C1—C6—C5	−177.6 (4)	N1—C7—C12—C11	177.1 (3)

### Hydrogen-bond geometry (Å, °)

**Table:**

*D*—H···*A*	*D*—H	H···*A*	*D*···*A*	*D*—H···*A*
N1—H1n···O2^i^	0.85 (3)	2.07 (2)	2.921 (3)	177 (5)

Symmetry codes: (i) −*x*+1/2, *y*−1/2, −*z*+1/2.

## References

[bb1] Brandenburg, K. (2006). *DIAMOND* Crystal Impact GbR, Bonn, Germany.

[bb2] Bruker (2007). *APEX2* and *SAINT* Bruker AXS Inc., Madison Wisconsin, USA.

[bb3] Farrugia, L. J. (1997). *J. Appl. Cryst.* **30**, 565.

[bb4] Gowda, B. T., Foro, S., Nirmala, P. G., Terao, H. & Fuess, H. (2009). *Acta Cryst.* E**65**, o877. 10.1107/S1600536809010459 PMC2969044 21582588

[bb5] Khan, I. U., Mariam, I., Zia-ur-Rehman, M., Arif Sajjad, M. & Sharif, S. (2010). *Acta Cryst.* E**66**, o1088. 10.1107/S160053681001322X PMC2979209 21579141

[bb6] Korolkovas, A. (1988). *Essentials of Medicinal Chemistry*, 2nd ed., pp. 699–716. New York: Wiley.

[bb7] Mandell, G. L. & Sande, M. A. (1992). In *Goodman and Gilman, The Pharmacological Basis of Therapeutics 2*, edited by A. Gilman, T. W. Rall, A. S. Nies & P. Taylor, 8th ed., pp. 1047–1057. Singapore: McGraw–Hill.

[bb8] Sharif, S., Iqbal, H., Khan, I. U., John, P. & Tiekink, E. R. T. (2010). *Acta Cryst.* E**66**, o1288. 10.1107/S1600536810016119 PMC2979509 21579386

[bb9] Sheldrick, G. M. (1996). *SADABS* University of Göttingen, Germany.

[bb10] Sheldrick, G. M. (2008). *Acta Cryst.* A**64**, 112–122. 10.1107/S0108767307043930 18156677

[bb11] Westrip, S. P. (2010). *J. Appl. Cryst.* **43**, 920–925.

